# Spatial epidemiology and genetic diversity of SARS-CoV-2 and related coronaviruses in domestic and wild animals

**DOI:** 10.1371/journal.pone.0260635

**Published:** 2021-12-15

**Authors:** Ariful Islam, Jinnat Ferdous, Md. Abu Sayeed, Shariful Islam, Md. Kaisar Rahman, Josefina Abedin, Otun Saha, Mohammad Mahmudul Hassan, Tahmina Shirin

**Affiliations:** 1 EcoHealth Alliance, New York, New York, United States of America; 2 Centre for Integrative Ecology, School of Life and Environmental Science, Deakin University, Victoria, Australia; 3 Institute of Epidemiology, Disease Control and Research (IEDCR), Dhaka, Bangladesh; 4 Department of Microbiology, University of Dhaka, Dhaka, Bangladesh; 5 Faculty of Veterinary Medicine, Chattogram Veterinary and Animal Sciences University, Chattogram, Bangladesh; Universite du Quebec a Montreal, CANADA

## Abstract

The Severe Acute Respiratory Syndrome Coronavirus 2 (SARS-CoV-2) showed susceptibility to diverse animal species. We conducted this study to understand the spatial epidemiology, genetic diversity, and statistically significant genetic similarity along with per-gene recombination events of SARS-CoV-2 and related viruses (SC2r-CoVs) in animals globally. We collected a number of different animal species infected with SARS-CoV-2 and its related viruses. Then, we retrieved genome sequences of SARS-CoV-2 and SC2r-CoVs from GISAID and NCBI GenBank for genomic and mutational analysis. Although the evolutionary origin of SARS-CoV-2 remains elusive, the diverse SC2r-CoV have been detected in multiple Rhinolophus bat species and in Malayan pangolin. To date, human-to-animal spillover events have been reported in cat, dog, tiger, lion, gorilla, leopard, ferret, puma, cougar, otter, and mink in 25 countries. Phylogeny and genetic recombination events of SC2r-CoVs showed higher similarity to the bat coronavirus RaTG13 and BANAL-103 for most of the genes and to some Malayan pangolin coronavirus (CoV) strains for the N protein from bats and pangolin showed close resemblance to SARS-CoV-2. The clustering of animal and human strains from the same geographical area has proved human-to-animal transmission of the virus. The Alpha, Delta and Mu-variant of SARS-CoV-2 was detected in dog, gorilla, lion, tiger, otter, and cat in the USA, India, Czech Republic, Belgium, and France with momentous genetic similarity with human SARS-CoV-2 sequences. The mink variant mutation (spike_Y453F) was detected in both humans and domestic cats. Moreover, the dog was affected mostly by clade O (66.7%), whereas cat and American mink were affected by clade GR (31.6 and 49.7%, respectively). The α-variant was detected as 2.6% in cat, 4.8% in dog, 14.3% in tiger, 66.7% in gorilla, and 77.3% in lion. The highest mutations observed in mink where the substitution of D614G in spike (95.2%) and P323L in NSP12 (95.2%) protein. In dog, cat, gorilla, lion, and tiger, Y505H and Y453F were the common mutations followed by Y145del, Y144del, and V70I in S protein. We recommend vaccine provision for pet and zoo animals to reduce the chance of transmission in animals. Besides, continuous epidemiological and genomic surveillance of coronaviruses in animal host is crucial to find out the immediate ancestor of SARS-CoV-2 and to prevent future CoVs threats to humans.

## Introduction

Coronaviruses (CoVs) appeared as public health threat during the emergence of the severe acute respiratory syndrome (SARS) during 2002–2003. It was the first pandemic in the 21^st^ century [1, 2]. The outbreak spread rapidly from southern China to almost 30 countries of the world. Within 6 months SARS caused greater than 8000 cases and 774 deaths in humans [2]. The causal agent of SARS was not known initially but later scientists identified the cause as a CoV. Scientists searched the source of the virus for so long and found that horseshoe bats harbor various SARS-CoV-related viruses [3, 4]. A CoV was detected in bats from China with 98% genetic identity to the SARS-CoV. Several recent investigations have revealed that the RaTG13 (China) and BANAL (Laos) CoVs identified in *Rhinolophus affinis* are the only near relatives of SARS-CoV-2 (horseshoe bats). Parts of their genetic coding, according to the researchers, also support allegations that COVID-19 is a natural virus [5]. Together with relatives of SARS-CoV-2 discovered in Thailand 2, Cambodia 3 and Yunnan, thus bats, may be the primary natural reservoir for the SARS-CoV and SARS-CoV-2 related viruses [5]. Moreover, the bat CoV can use the angiotensin converting enzyme 2 (ACE2) receptor on human cells and can infect them [6]. Not only in humans but severe CoV outbreaks in animals were also reported [7]. For example, piglets suffer from swine acute diarrhea syndrome (SADS) severely in China in 2002 [8]. The SADS CoV was also traced back to a nearby colony of the bat. The bat CoV HKU2, found in *Rhinolophus* spp., has greater than 98% genetic relation with that of SADS-CoV [8].

After that, the world again observed the emergence of a CoV during 2012. The virus was named middle east respiratory syndrome CoV (MERS-CoV). It was also a beta CoV like SARS, infected 2249 humans in 27 countries [9]. Though camels were identified as a reservoir host of the virus, it was evident that this virus was also originated from bats [10–12]. Most recently, the Severe Acute Respiratory Syndrome Coronavirus 2 (SARS-CoV-2) has been reported in China in December 2019 [13]. The virus was thought to be originated from an animal market in Wuhan. Different animal species like poultry, snakes, hedgehogs, etc. were traded at that time [14]. Not only live animals, meat, and carcasses were also present in that market at that time [15]. The exact precursor has not been established yet, but later studies pointed out that *Rhinolophus* bats have a similar kind of CoV [16]. Though SARS-CoV-2 has a 96% genetic resemblance with Horseshoe bat (*R*. *affinis*) CoV RaTG13, but no natural infection of SARS-CoV-2 in bat has been reported [17–20]. Two bat species namely *R*. *affinis* and *R*. *malaynus* can be the ancestral reservoir of SARS-CoV-2. But these bat species cannot transmit SARS-CoV-2 directly to humans [16].

After the onset of the pandemic by SARS-CoV-2, the human to animal transmission of the virus have been reported in pet, farmed, zoo, and wild animals [21]. Cat and dog who were in contact with the infected human have been identified SARS-CoV-2 positive. So, there is occasional spill-over evidence from human to animal species [22]. Experimental studies showed that several animal species are susceptible to the SARS-CoV-2 such as, ferrets, cats, hamsters, and rhesus macaques [23]. Gradually, different variants e.g., GRY (B.1.1.7)/α variant [24] and mink variants [25] have also been detected in animal populations. Therefore, animal hosts may play a very important role in the emergence of new CoVs at regular intervals. Thus, we conducted the present study to understand the spatial epidemiology and genetic characterization of SARS-CoV-2 in domestic and wild animals globally.

## Materials and method

### Spatial epidemiology of SARS-CoV-2 and related CoV

We collected SARS-CoV-2 animal infection metadata from the World Organization for Animal Health (OIE) [26], the Global Initiative on Sharing All Influenza Data (GISAID) [27], and the United States Department of Agriculture (USDA) [28] (Table 1). We collected animal species and the number of animals infected in different countries by SARS-CoV-2 and SARS-CoV-2 related viruses. We presented the spatial distribution of SARS-CoV-1 and SARS-CoV-2 related viruses in bat species and natural infection of SARS-CoV-2 in domestic and wild animal species on the world map using the world’s shapefile (www.tapiquen-sig.jimdofree.com) in ArcGIS software [29, 30].

**Table 1:** Distribution of SARS-CoV-2 related viruses in bats and pangolins.

Animals	Species	Virus name	Sampling year	Country
Bat	*Rhinolophus blythi*	PrC31	2018	Yunnan, China
	*R*. *sinicus*	RsYN03	2019	China
	*R*. *stheno*	RsYN04, 09	2020	China
	*R*. *pusillus*	RpYN06	2020	China
	*R*. *malayanus*	RmYN01, 02, 05, 07, 08	2019–20	China
	*R*. *affinis*	RaTG13	2013	China
	*R*. *shameli*	RShSTT182, 200	2010	Cambodia
	*R*. *cornutus*	Rc-o319	2013	Japan
	*R*. *acuminatus*	RacCS203	2020	Thailand
	*R*. *marshalli*	BANAL-236	Jul 2020- Jan, 2021	Northern Laos
	*R*. *pusillus*	BANAL-27, 103		
	*R*. *malayanus*	BANAL-52, 116, 242, 247		
Pangolin	*Manis javanica*	Pangolin coronavirus	2017 and 2019	China
	*M*. *pentadactyla*	Pangolin coronavirus	2017	China
	*M*. *javanica*	Pangolin coronavirus	2017	Thailand

### Genomic epidemiology of SARS-CoV-2 and related CoV

#### Retrieval of viral sequences

We retrieved the complete genome sequences with an average length of more than 29000 base pairs, high coverage, metadata, and annotations of SARS-CoV-2 and SARS-CoV-2 related viruses from GISAID [27] (S1 Table) and National Centre for Biotechnology Information (NCBI) GenBank database [31].

#### Phylogenetic analysis

We performed the multiple sequence alignment using a virus pathogen resource (https://www.viprbrc.org/) because of the sizeable sequential data set. Multiple sequence alignments were finally opened with Molecular Evolutionary Genetics Analysis (MEGA 7.0) computing platforms to remove all ambiguous and low-quality sequences. We prepared a phylogenetic tree according to the neighbor-joining method using MEGA 7.0. In this method, the likelihood is calculated for each nucleotide substitution in the alignment. This is the most computationally intensive but flexible method for determining topology and branch lengths. It renders the statistical model for evolutionary diversity that varies across branches.

#### SimPlot similarity analysis

Using the Wuhan SARS-CoV-2 2020 genome as a reference, SimPlot v3.5 [32] was used to perform a sliding window analysis and find patterns of sequence similarity. In addition, the RatTG13 genome was compared to the Guangxi Pangolin CoV group, Guangdong Pangolin CoV group, Bat Laos BNAL CoV group, Bat Cambodia RShSTT CoV group, and Bat China RpYN CoV group consensus genomes (S1 Table). SimPlot v3.5 constructed these consensus genomes as the default consensus genomes for representing a collection of species. In addition, gene-by-gene SimPlot similarity analysis was used to compare the genes of the Wuhan SARS-CoV-2 2020 reference genome to the genes of the reference sequences mentioned above.

Individual genes and whole genome sequences of the Wuhan SARS-CoV-2, RaTG13, GX Pangolin CoV, Gu Pangolin CoV, Bat BANAL-103, Bat RShSTT200, and Bat RpYN06 were subjected to the Φ-recombination test [33] to discover recombination patterns (S1 Table). The -test was carried out with sliding windows ranging in size from 50 to 200 square feet and a window progress step of 1. Martin [34] offered the version (RDP5: Recombination detection program version 5) of the Φ-test that we utilized in our investigation.

#### Mutational analysis

We used the Swiss model repository (https://swissmodel.expasy.org/repository/species/2697049) of SARS-CoV-2 and GISAID to know the location of the amino acid’s position of each protein of the SARS-CoV-2 genome. Furthermore, mutations that result in amino acid substitutions were investigated using the Wuhan reference sequence (NC 045512.2) and the GISAID platform, which included the CoVserver enabled by GISAID in the GISAID EpiCoV data base, as well as a blast (https://blast.ncbi.nlm.nih.gov/) of the entire genome and individual proteins [35].

## Results

### Spatial distribution of SARS-CoV-2 related coronaviruses (SC2r-CoVs) in horseshoe bats and pangolin

**Fig 1:**
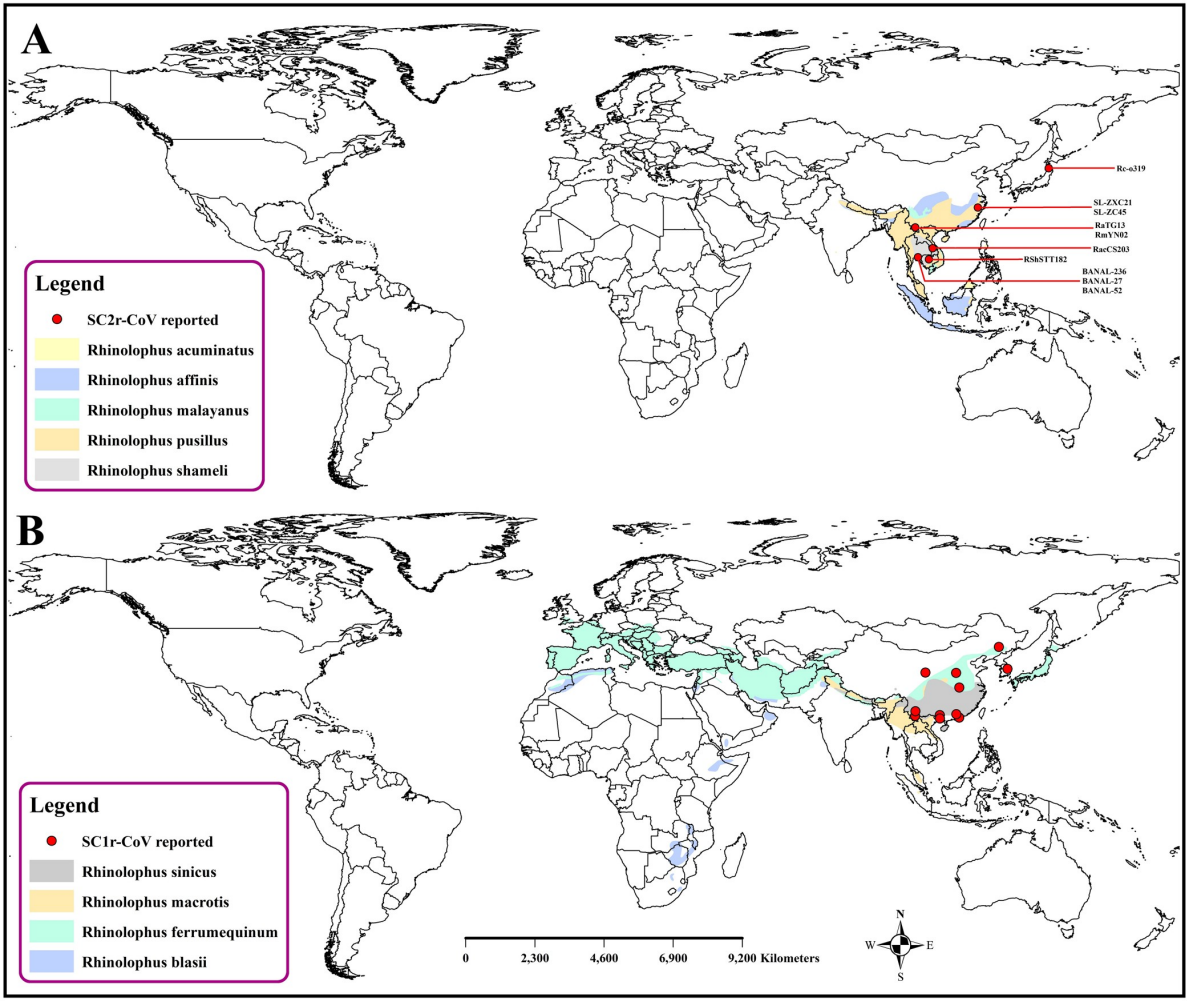
Spatial distribution of SARS-CoV-2 related viruses reported in horseshoe bat species in the world. A. SC2r-CoV reported in bat species in the world; B. SC1r-CoV reported in bat species in the world.

SARS-CoVs related viruses have been identified in horseshoe bats from China, Thailand, Cambodia, and Japan (Fig 1). All these viruses have been found in samples collected between 2010 to 2020. In China, *Rhinolophus sinicus*, *R*. *stheno*, *R*. *pusillus*, *R*. *malayanus*, and *R*. *affinis* harbor SARS-CoV-2 like viruses. The virus RpYN06 from *R*. *pusillus* was the most closely related to SARS-CoV-2.

The more recent detection has been in Japan and Cambodia which are closely related to SARS-CoV-2. This is the first known SARS-CoV-2 related virus found outside China. In Cambodia, the virus was found in two Shamel’s horseshoe bats (*R*. *shameli*) which were captured back in 2010. Another virus called Rc-o319 was identified in Japanese horseshoe bat’s (*R*. *cornutus*) frozen droppings collected in 2013. Finally, the SARS-CoV-2 like virus was detected in *R*. *acuminatus* bats in Thailand. Similarly, SARS-CoV-2 viruses have also been detected in two dead Malayan pangolins (*Manis javanica*) from China. Moreover, antibodies against SARS-CoV-2 were detected in a pangolin at a wildlife checkpoint in southern Thailand. Recently, the closest relative of SARS-CoV-2 have been detected in cave bats in Northern Laos (Table 1).

### Spatial epidemiology of SARS-CoV-2 infection in domestic and wild animals

#### Possible evolution and transmission pathways of SARS-CoV-2 and its related virus at the animal-human interface

**Fig 2:**
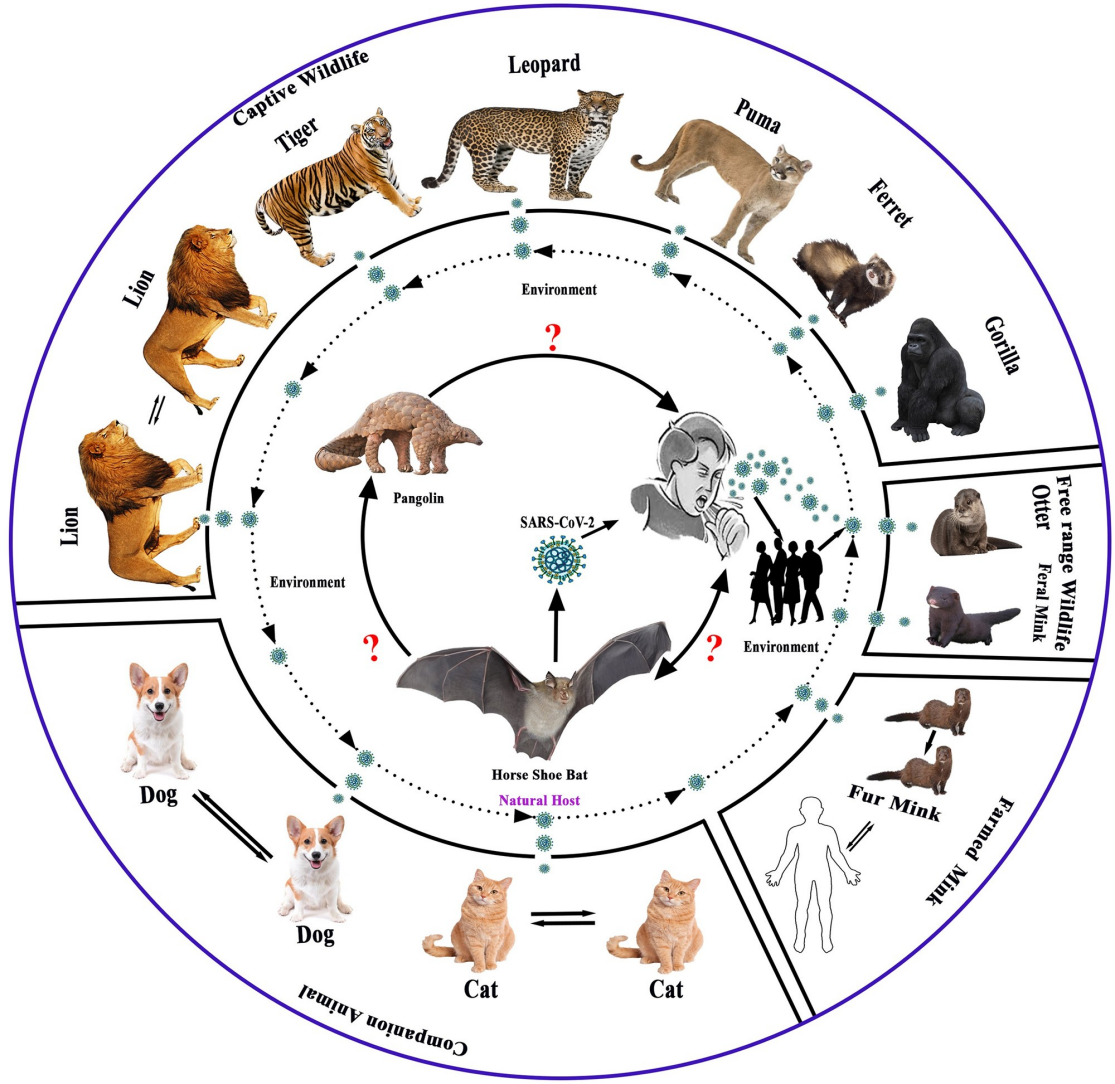
Possible evolution and transmission pathways of SARS-CoV-2 and its related virus at animal-human- ecosystem interface.

Natural infection of SARS-CoV-2 has been identified in different animal species (Fig 2), i.e.; cat, dog, mink, cougar, gorilla, lion, snow leopard, tiger, puma, ferret, and otter in 25 countries (Table 2). The countries reported animal species infected with SARS-CoV-2 are summarized below-

**Table 2:** Natural infection of SARS-CoV-2 in animal.

Animal species	No. of the country affected	No. of the affected animal
Domestic Cat (*Felis catus*)	30	156
Dog (*Canis lupus familiaris*)	7	113
Tiger (*Panthera tigris*)	2	15
Lion (*Panthera leo*)	4	22
Gorilla (*Gorilla gorilla*)	2	3
Leopard cat (*Prionailurus bengalensis euptilurus*)	1	3
Snow leopard (*Panthera uncia*)	1	4
Puma (*Puma concolor*)	2	2
American Mink (*Neovison vison*)	18	6590 and 69 farms in the Netherlands
Domestic Ferret (*Mustela furo*)	1	1
Cougar (*Puma concolor*)	1	1
Asian small-clawed otter (*Aonyx cinereus*)	1	1
Beavers (*Castor canadensis*)	1	7
Wild white-tailed deer (*Odocoileus virginianus*)	1	8

SARS-CoV-2 can produce subclinical infection in cats. Natural infection in the cat has been reported in different countries including Spain, China, Hong Kong, Belgium, the USA, France, Germany, Russia, and the UK. On the other hand, Hong Kong, the USA, Germany, Japan, Canada, Brazil, Argentina, Mexico, Bosnia & Herzegovina reported SARS-CoV-2 in dogs. Furthermore, alpha (α) variant of concern (VOC) (lineage B.1.1.7) has been identified in a pet cat after contacted with infected COVID-19 owner in the USA [36]. The alpha VOC was also detected in cats from Thailand and Italy [24]. And most recently in a zoo in Hyderabad, India, eight Asiatic lions are found to be infected by delta variant, (B.1.617.2).

#### Global distribution of SARS-CoV-2 infection in domestic and wild animals

**Fig 3:**
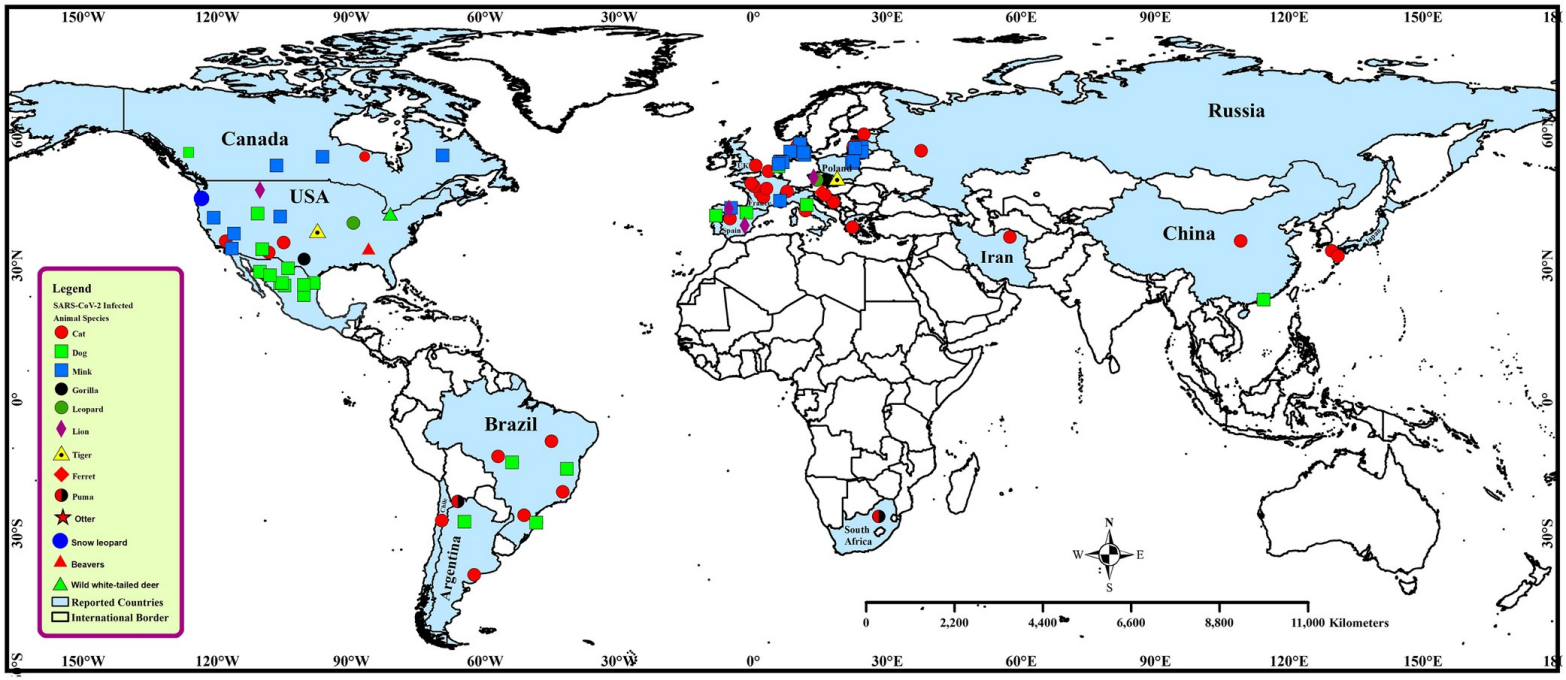
Spatial distribution of SARS-CoV-2 in domestic and wild animals in the world.

To date, SARS-CoV-2 has been identified in mink (*Neovison vison*) farms from Denmark, the Netherlands, France, Spain, Sweden, Canada, Greece, Lithuania, Poland, Latvia, Italy, and the USA [37]. Till October 25, 2020, 69 infected mink farms were identified in the Netherlands. Besides, till December 1, 2020, approximately 20% (289 farms) of all mink farms in Denmark have been infected with COVID-19 [37] (Fig 3).

### Genomic epidemiology of SARS-CoV-2 and its related viruses in domestic and wild animals

#### Phylogenetic analysis of SC2r viruses in bats and pangolins

SC2r-CoVs has a 96.10% genetic resemblance with Horseshoe bat (*R*. *affinis*) CoV RaTG13. SC2r viruses from bats from China formed different clusters depending on their genetic relatedness. SC2r viruses from Thailand and Cambodia separately formed two distinct clusters by themselves. Bat CoVs, RmYN01, and RmYN07 from 2019 and 2020 were genetically similar and grouped together. Another important finding was SC2r viruses from *R*. *pusillus* from China, 2015 and 2017 showed nucleotide similarities. Moreover, another bat CoV cluster was formed distinctly by RmYN05 and RmYN08 virus from *R*. *malayanus* from China, 2020 and by Rc-o319 virus from *R*. *cornutus* from Japan, 2013.

**Fig 4:**
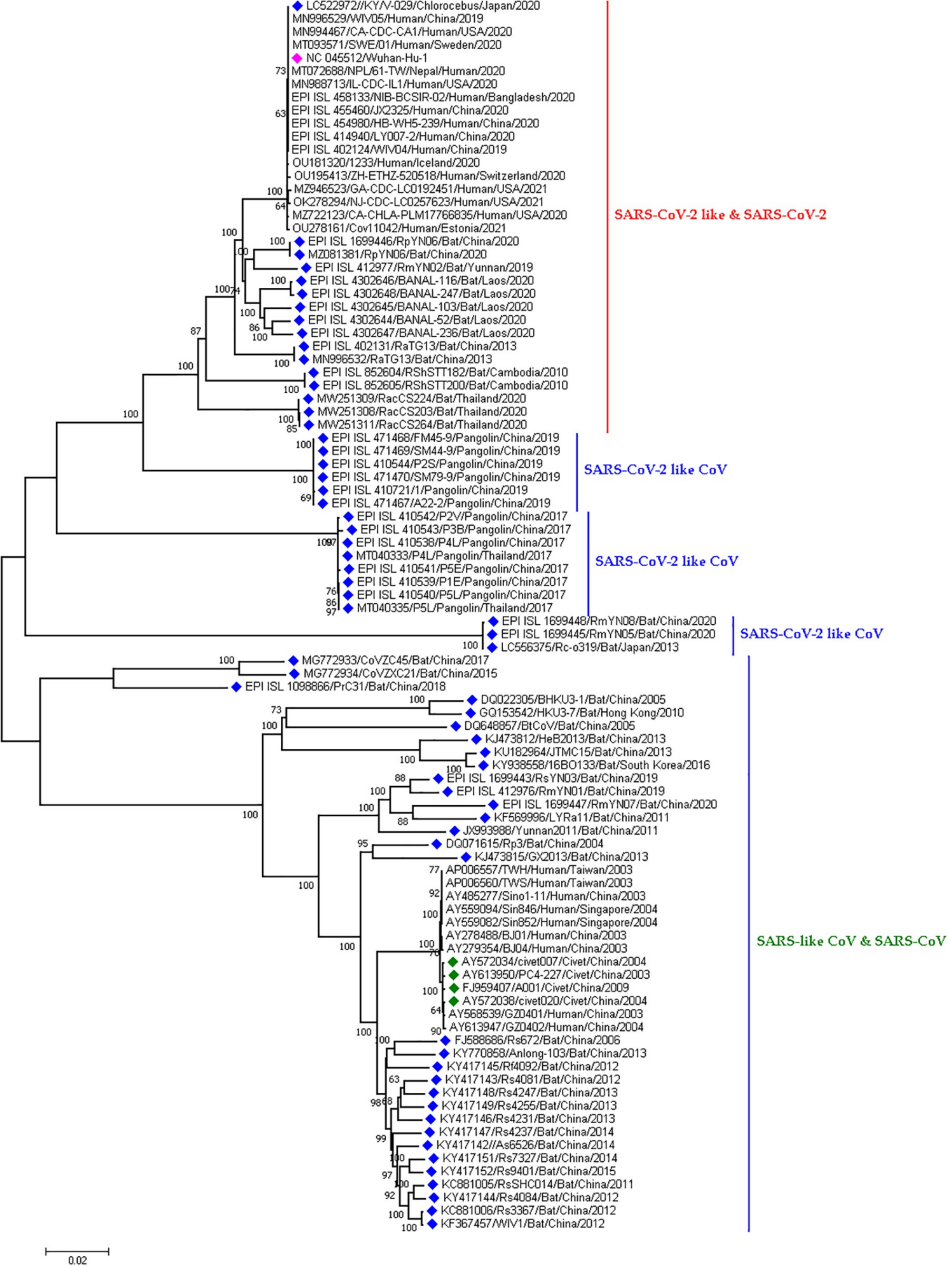
Phylogenetic analysis of SARS-CoV-2 related viruses retrieved from bat and pangolin. Here, blue dots denote SARS-like CoV, SARS-CoV-2 and SARS-CoV-2 like CoV viruses, green and pink dotes denotes SARS-CoV and Wuhan-Hu-1 viruses respectively.

Moreover, SC2r pangolin coronaviruses from Guangxi, China, and Thailand from 2017 were grouped in a cluster separately from other pangolin coronaviruses. Another pangolin coronavirus cluster was found to consist of samples from Guangdong, China in 2019 and this cluster is closely related to the reference strain of human SARS-CoV-2 from Wuhan (Fig 4).

#### Phylogenetic analysis of SARS-CoV-2 in domestic and wild animals

**Fig 5:**
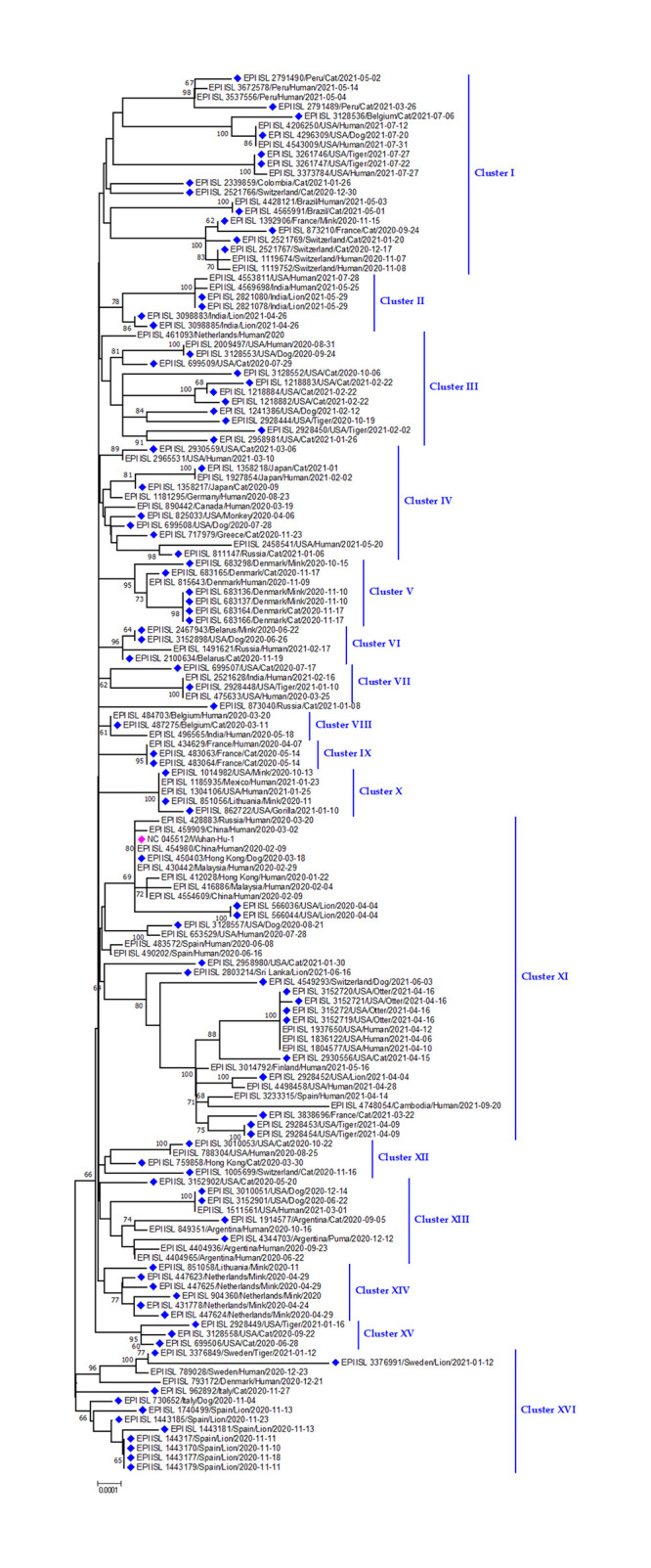
Phylogenetic analysis of SARS-CoV-2 sequences from domestic and wild animals. Blue dots denote sequences detect in domestic and wild animals. Pink dots = indicate Wuhan-Hu-1 sequence.

Phylogenetic analysis of SARS-CoV-2 sequences from different animals proved their relation to human sequences from the same region at the same period. All the animal strains have similarities with human strains (Fig 5). The SARS-CoV-2 was found in gorillas, dog, cat, monkey, otter, lions, leopards, and tigers in the various portion of the world (Peru, France, Cambodia, USA, Belgium, India, Sweden, Italy, Spain, Japan, Greece, Denmark, Russia, Netherlands) and clustered together showing genetic relatedness (Fig 5). Moreover, this cluster has similarities with human strains from the responsible countries. A similar relationship was detected among dog, tiger and cat strains (Cluster III) of SARS-CoV-2 in the USA. Moreover, SARS-CoV-2 in Lion in Spain was found to be related to Cat & Dog strain from Italy. Interestingly, SARS-CoV-2 in Lion in India was also found to be related to human strain form USA (Fig 5).

**Fig 6:**
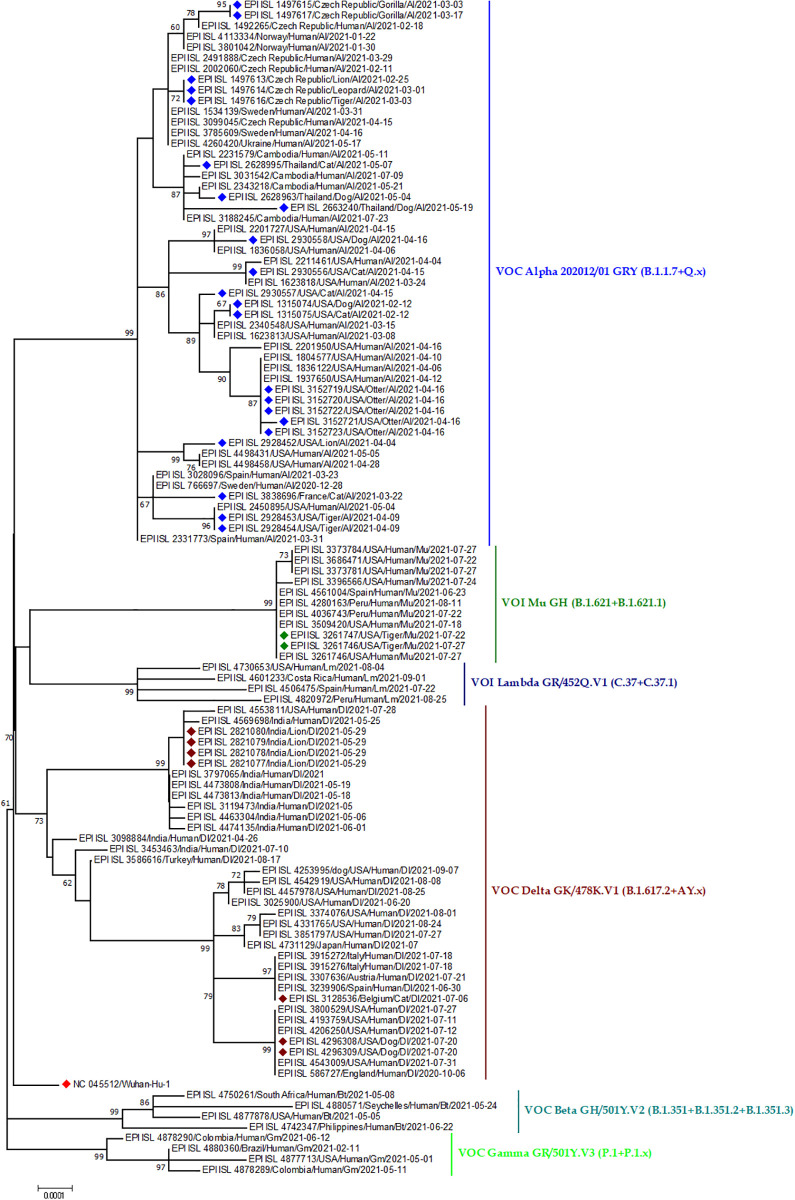
Phylogenetic analysis of SARS-CoV-2 and representative sequences of alpha, beta, delta, lambda, mu, gamma CoV sequences. Blue dots denote Alpha sequences, lime (Gamma), maroon (Delta), teal (Beta), lambda (Navy), green (Mu). Red dots indicate Wuhan-Hu-1 sequence.

Not only the pango lineage of SARS-CoV-2 isolates but also the VOC strains in the world have also formed a cluster in the phylogeny (Fig 6). In case of Alpha variant, Gorilla strain (VOC Alpha 202012/01 GRY (B.1.1.7 +Q.x)) from Czech Republic and human strain from the others few European countries like Norway, Sweden and Ukraine had nucleotide resemblance. Even the cat strain from the France, tiger strain from USA has genetic relations with humans of the respective region. Similarly, cat and human strains from USA were found to group together. A large cluster was formed by sequences from Otter of USA and human of USA. (Fig 6). Similarly, in case of Delta variants, lion strain from the India have found genetic similarity with the B.1.627.2 sequences from the other Indian patients. Whereas the cat delta variant reported from Belgium showed a genetic relation to human in the USA. Finally, evolutionary pattern of Mu variant (VOI Mu GH (B.1.617.2 + B.1.621.1)) also expressed the similar types of genetic relatedness as other variants (Fig 6).

#### Phylogenetic analysis of SARS-CoV-2 in farmed minks

**Fig 7:**
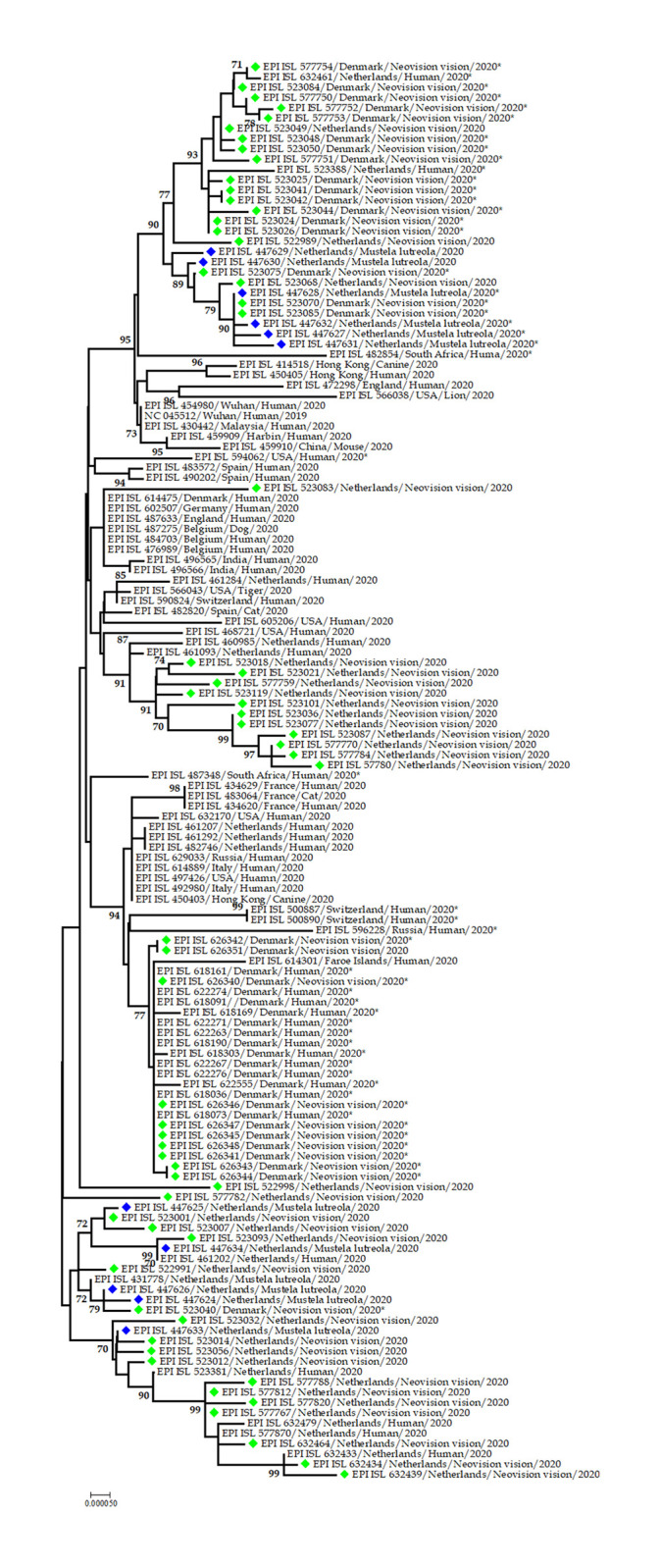
Phylogenetic analysis of SARS-CoV-2 sequences from minks. Lime dots denote sequences from American mink whereas blue dots denote sequences from European mink. Star (*) indicates mink variant mutation in spike protein (Spike_Y453F) reported in mink and humans.

The phylogenetic analysis of SARS-CoV-2 sequences from mink from around the world showed that the mink strains are genetically closely related to each other. Mink sequences are mainly from Denmark and Netherlands, and a few from Belarus, Lithuania, and Poland. Some human strains were also found within the mink clusters. SARS-CoV-2 from American mink and European mink created two separate clusters in the phylogenetic tree. SARS-CoV-2 strains of American mink from Denmark and Netherlands resemble human strains from the Netherlands. Mink variant mutation in spike protein (spike_Y453F) was more common in SARS-CoV-2 strains from American mink than that of European mink. Mink mutations were found in human strains from the Netherlands, South Africa, USA, Denmark, Russia, Switzerland, England, and Poland. Even cats were also found to have the mink mutation in Denmark (Fig 7).

#### Similarity analysis of SARS-CoV-2

**Fig 8:**
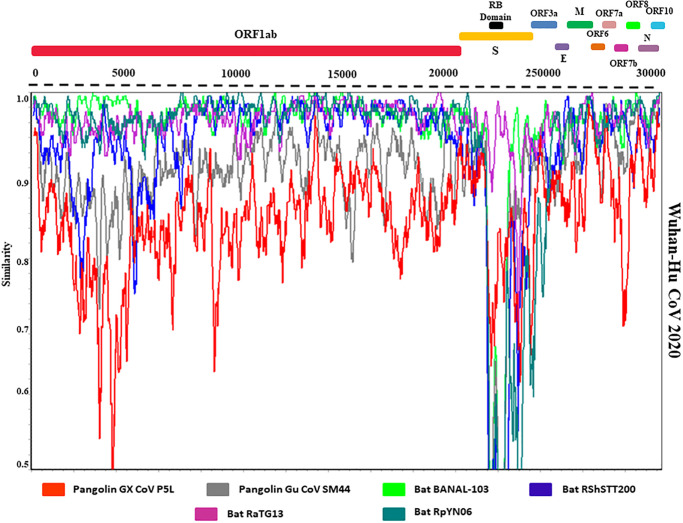
SimPlot was used to compare the genomes of SARS-CoV-2 and comparable viruses. The RatTG13 CoV genome (green) and the consensus genomes of the GX Pangolin CoV (red), Gu Pangolin CoV (gray), Bat BANAL-103 (lime), Bat RShSTT200 (blue), Bat RaTG13 (violet) and Bat RpYN06 (teal) groups are shown beside the Wuhan SARS-CoV-2 2020 reference genome. The RB domain, as well as gene limitations for ORF1ab, S, ORF3a, E, M, ORF6, ORF7a, ORF7b, ORF8, N, and ORF10, is displayed at the top of the image. Distinct colors reflect different groups of sequences integrated in SimPlot analysis, corresponding to species groupings in the entire genome phylogeny depicted in panel.

The Wuhan SARS-CoV-2 and RaTG13 genomes have 96.14 percent whole-genome similarity, whereas the Wuhan SARS-CoV-2 and Gu Pangolin genomes are 89.14 percent identical, according to our SimPlot study (Fig 8) of 21 CoV genomes. Surprisingly, both bat CoV of Laos and Cambodia genomes exhibit more than 96 percent similarity. The RaTG13, RShSTT, and BANAL bat CoV genomes are the most similar to the SARS-CoV-2 genome by a long shot. The Wuhan SARS-CoV-2 and GX Pangolin CoV genomes, for example, have just 85.43 percent whole-genome identity. Bat is a plausible reservoir of origin for SARS-CoV-2, as it was during earlier CoV epidemics, due to the striking similarities between SARS-CoV-2 and RaTG13, RShSTT, and BANAL (Fig 8).

**Fig 9:**
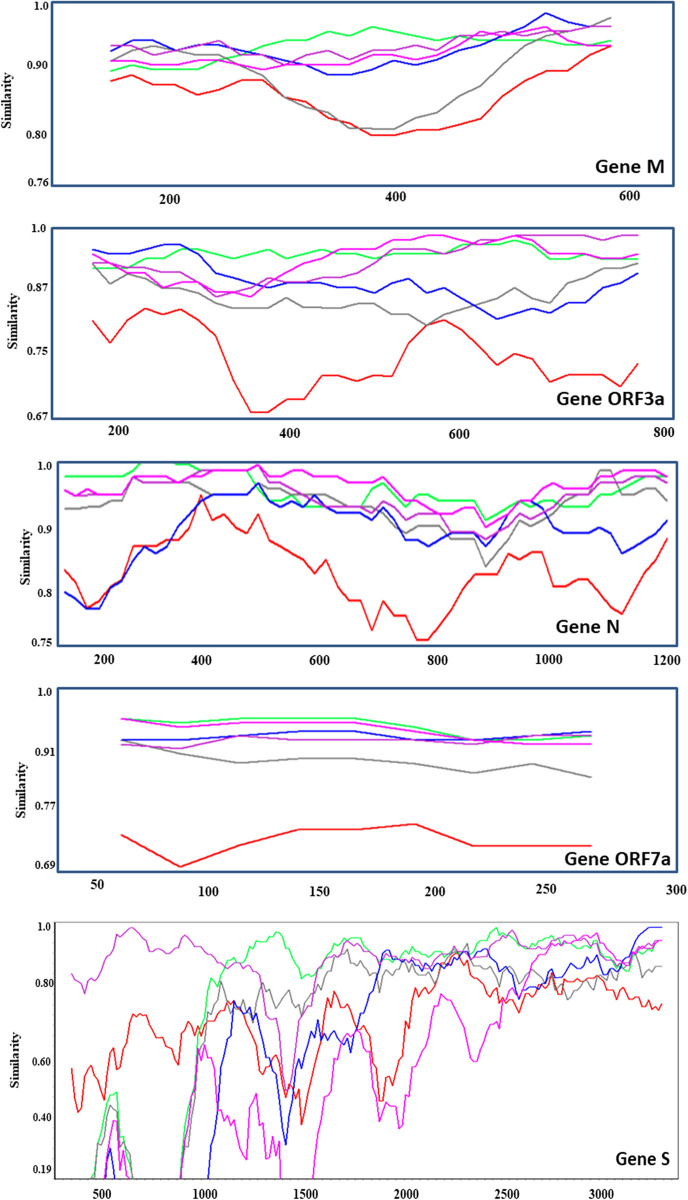
Gene-by-gene SimPlot similarity analysis of the Wuhan SARS-CoV-2 2020 reference genome against the GX Pangolin CoV (red), Gu Pangolin CoV (gray), Bat BANAL-103 (lime), Bat RShSTT200 (blue), Bat RaTG13 (violet), and Bat RpYN06 (pink) consensus genomes. Similarity plots are shown for the genes ORF1ab, M, ORF3a, N, ORF7a, and S, showing the most significant overlaps between the sequences as mentioned above.

The Wuhan SARSCoV-2 gene sequences were compared to the RaTG13, Gx Pangolin CoV, Gu Pangolin CoV, Bat BANAL, Bat RShSTT, and Bat RpYN group sequences using a thorough similarity analysis at the gene level (Fig 9). According to our gene-by-gene study, N sections of SARS-CoV-2 were more similar to Gu Pangolin CoV than to Gx. Furthermore, we discovered that the SARS-CoV-2 gene sequences are substantially more similar to those of bat viruses than those of Pangolin CoVs, and occasionally even RaTG13, in several continuous gene areas, notably genes ORF1ab, ORF3a, S, ORF7a, M, and N (Fig 9).

#### Φ-test recombination analysis

To study possible recent recombination events between orthologous gene sequences of the Wuhan SARS-CoV-2, RaTG13, Gx Pangolin CoV, Gu Pangolin CoV, Bat BANAL-103, Bat RShSTT200, and Bat RpYN06 viruses, we did gene-by-gene and whole genome-test recombination analyses. The test was conducted with several sliding window sizes ranging from 50 to 200 (with a step of 50) and a window progress step of 1, because the window size can influence the test outcome. Table 3 shows the window size findings that correspond to the least p-value found for a specific gene. Significant p-values were defined as those less than or equal to the 0.05 threshold. They show that recombination is present in the gene under investigation. Statistically significant recombination events involving these six coronaviruses have been discovered in genes ORF1ab, S, N, and entire genome sequences, according to the test (Table 3). Recombination events in the genes ORF1ab and S were discovered with high confidence, with p-values of 3.381×10^−2^ and 5.128332×10^−2^, respectively.

**Table 3:** The recombination test outcomes for Wuhan SARS-CoV-2, RaTG13, Pangolin Gx CoV P5L, Pangolin Gu CoV SM44, Bat BANAL-103, Bat RShSTT200, and Bat RpYN06 gene and complete genome sequences.

Region	Φ-test result (p-value)	Recombination detected (Yes/no)	Window size
Gene ORF1ab	3.381 × 10^−2^	Yes	200
RB domain	Too short	-	-
Gene S	5.128332 × 10^−4^	Yes	150
Gene ORF3a	0.3028	No	200
Gene E	0.186	No	200
Gene M	0.3594	No	150
Gene ORF6	Too short	-	-
Gene ORF7a	0.01538	NO	200
Gene ORF7b	Too short	-	-
Gene ORF8	0.06322	No	200
Gene N	0.68714	Yes	100
Gene ORF10	Too short	-	-
Whole genomes	0.066100	Yes	200

#### Clade and lineage diversity of SARS-CoV-2 within and among domestic and wild animals

**Fig 10:**
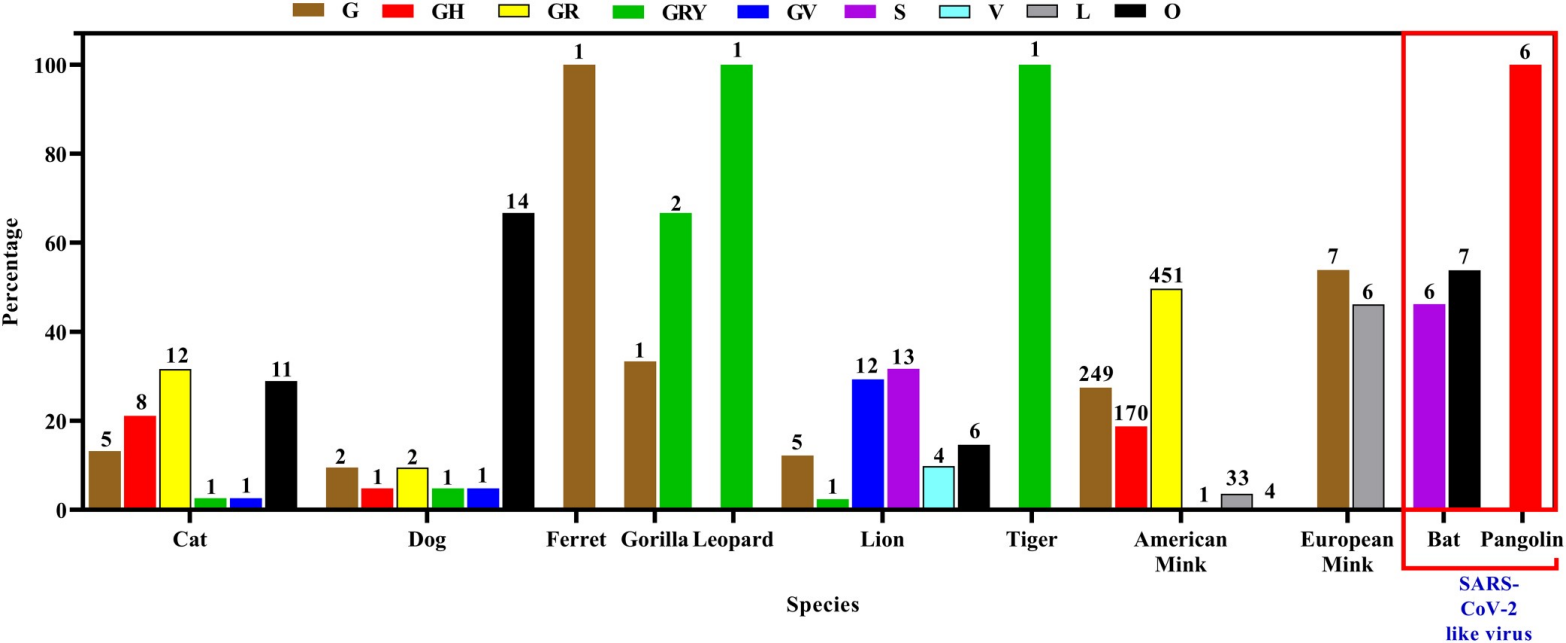
Clade diversity of SARS-CoV-2 in different animal species.

Bat strains were found to be under lineage A.3, A.21, A.23, B.1.177, B.4, and clade O, S. On the other hand, Malayan pangolin strains belong to lineage A, B.5, and B.15, clade O and S. Fig 10 illustrates the overall species variation under each clade. The α variant under clade GRY was found in cat, dog, gorilla, leopard, lion, and tiger (Fig 10).

On contrary, clade O of SARS-CoV-2 was most common (66.7%) in the dog but other clades like G, GH, GR, GRY, and GV were also detected. In the cat, clade GR, O, and GH were found at 31.6%, 28.9%, and 21.1% respectively though G, GRY, and GV clades were found. American minks and European minks were found to be infected mostly by clade GR (49.7%) and clade G (53.9%), respectively, but a reasonable percentage was also affected by clade GH and L. The lion infected mainly by clade GV (54.5%), G (22.7%), and V (18.2%). In the tiger, we found mainly clade GH (85.7%) and GRY (14.3%) whereas in gorilla 66.7% were under clade GRY and 33.3% were under clade G. In ferret and leopard cat, clade G and GRY were found, respectively (S1 Fig).

**Fig 11:**
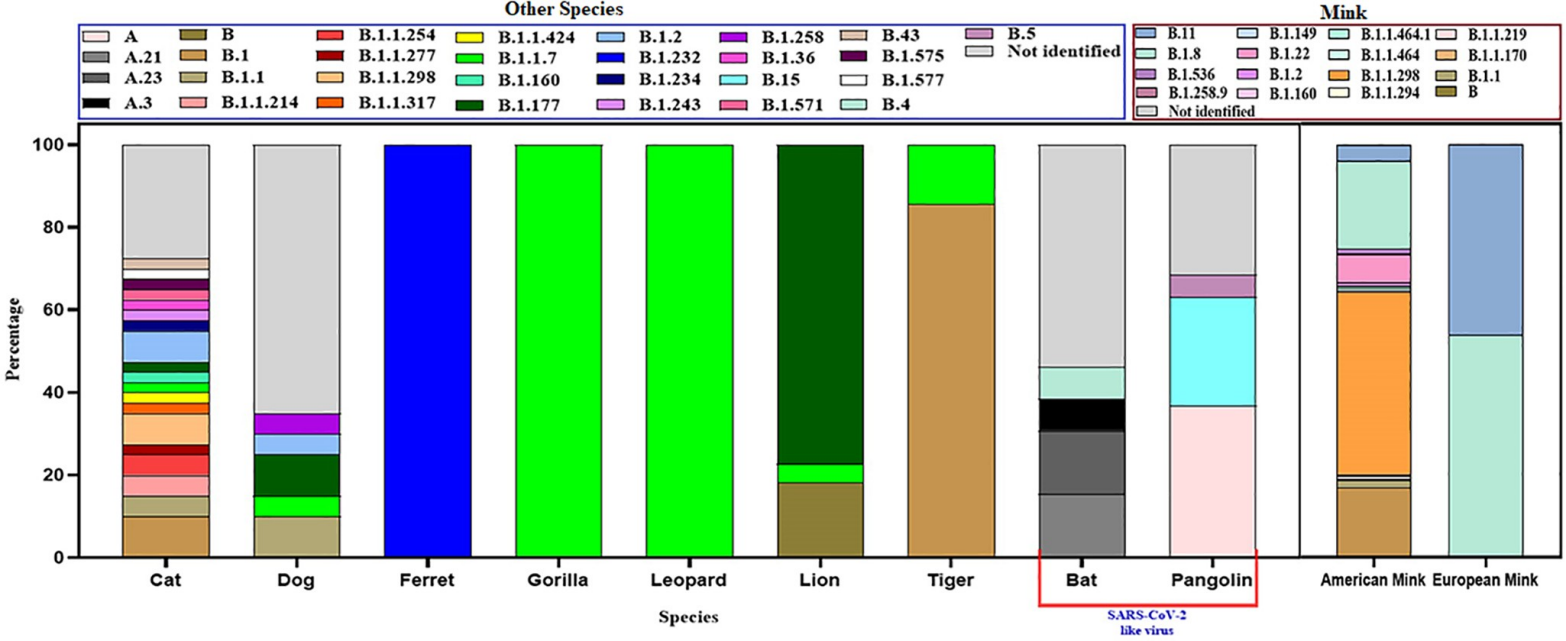
Lineage diversity of SARS-CoV-2 among different animal species.

B.1.1, B.1.1.7, B.1.177, B.1.2, B.1.243, B.43 lineages were found in the dog. The α variant (B.1.1.7) was found in 4.8% of dogs. In the cat, the predominant lineage type was B.1 (10.5%) and α variant lineage B.1.1.7 prevalence was 2.6%. Only 3 lineage- B, B.1.1.7, and B.1.177 were detected in lions among which B.1.177 were found in the highest percentage (77.3%). On the other hand, the sequences identified were mostly under lineage B.1 (85.7%) in tiger, whereas α variant (B.1.1.7) was found as 14.3%. The α variant was also detected in leopard cats but in ferret B.1.258 lineage was identified. In gorilla, 66.7% were α variant and the rest was under lineage B.1.232. Highest lineage diversity identified among mink. Mink strains were mostly under lineage B.1.1.298 (43.8%) followed by B.1.8 (21.7%), B.1 (16.5%), B.1.22 (6.7%) (Fig 11).

In Fig 11, we showed the abundance of specific lineage among different animal species. The α variant (B.1.1.7) was detected in dogs, cat, gorilla, lion, tiger, and leopard cat. The highest percentage was found in gorilla at 28.6%.

#### Mutational patterns of SARS-CoV-2 in intra and interspecies of animals

**Fig 12:**
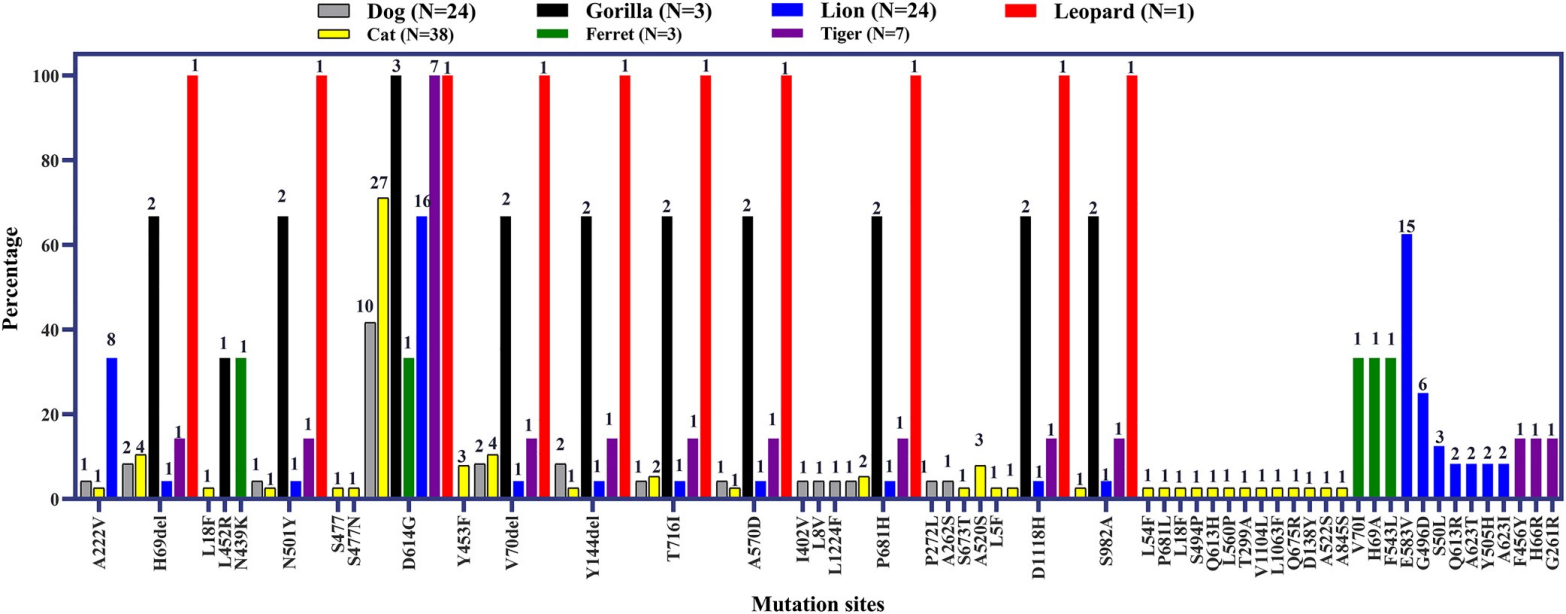
Frequency of specific mutation points at the amino acid level in spike protein of SARS-CoV-2 in different animal species.

In the dog, cat, gorilla, lion, and tiger, Y505H and Y453F were the most common mutations followed by Y145del, Y144del, and V70I in S protein. These mutations were also found in ferret and leopard cat (Fig 11). Some mutations in S protein like L54F, P681L, L18F, S494P, Q613H, L560P, T299A, V1104L, L1063F, Q675R, D138Y, A522S, A845S were only detected in cat, not in any other animal species. Similarly, lions have some unique mutations like E583V, G496D, S50L, Q613R, A623T, Y505H, A623I (Fig 12).

**Fig 13:**
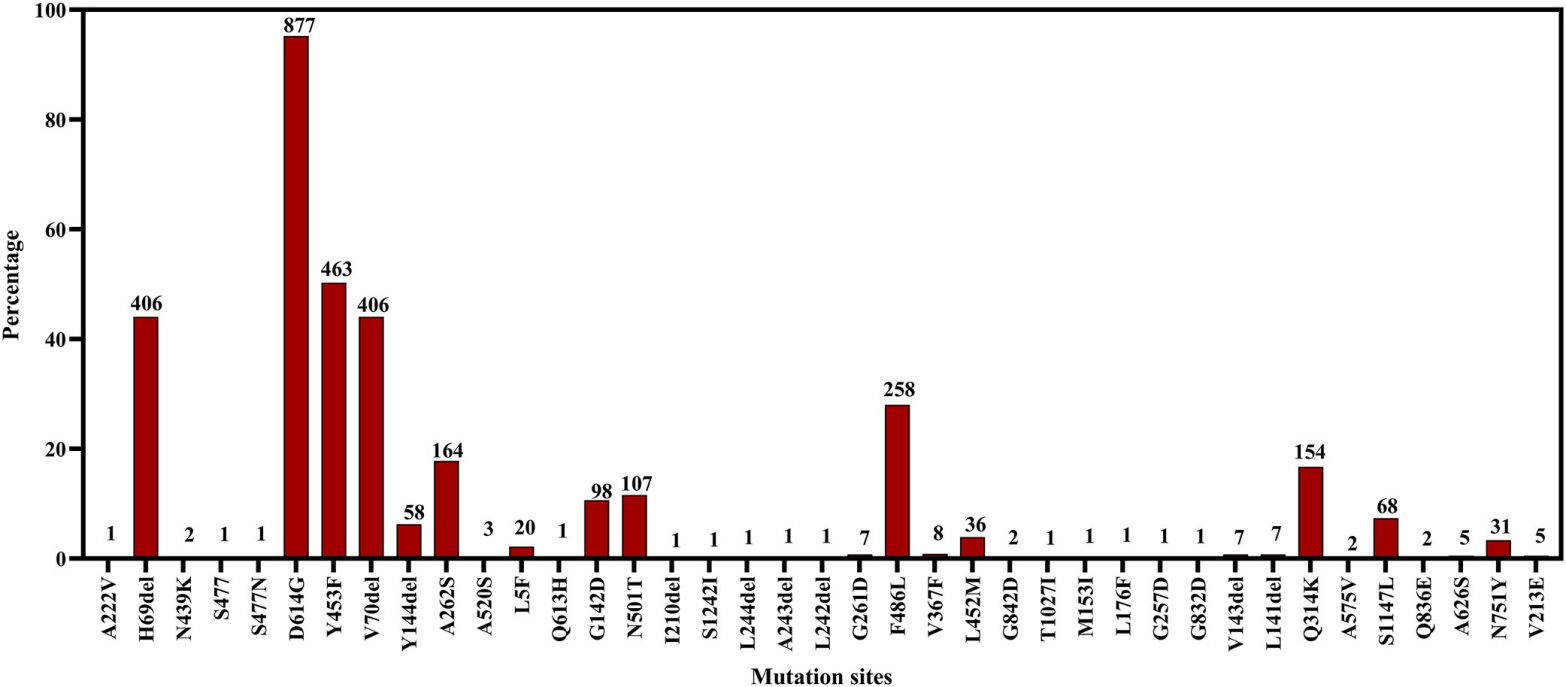
Frequency of specific mutation points at the amino acid level in spike protein of SARS-CoV-2 in American mink.

The highest mutations were found in mink among all the animal species. The D614G in spike protein and P323L in non-structural protein 12 (NSP12) were the most prevalent (95.2%) mutations in minks. Other commonly found mutations were Y453F (50.3%), S194L (49.4%), R203K (49.1%), and G204R (47.6%) in N protein of mink (Fig 13).

**Fig 14:**
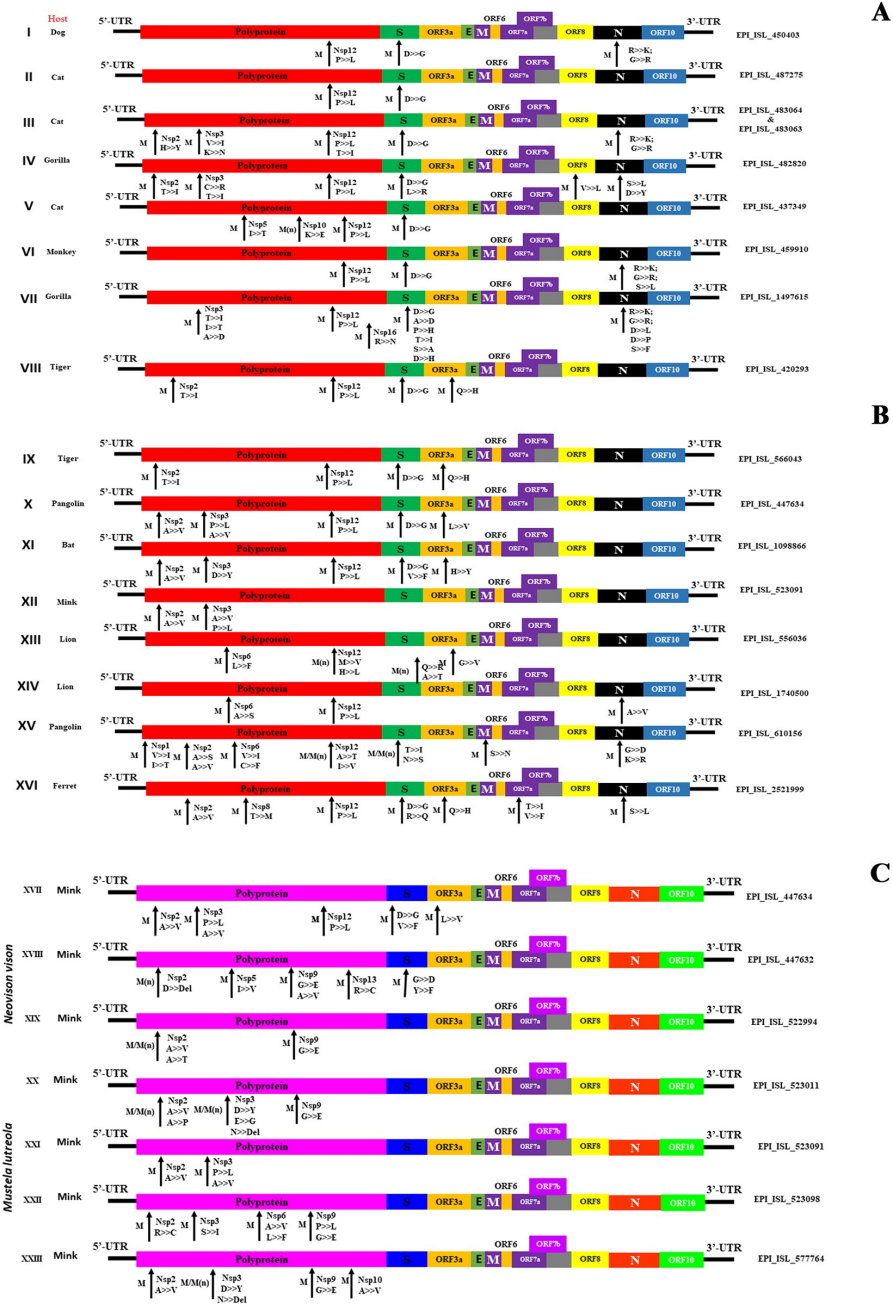
Genomic mutation analysis of animals originated SARS-CoV-2. Genomic mutation analysis of SARS-CoV-2 strains identified. >> ↑ and marking indicating the mutational changes among 12 SARS-CoV-2 (EPI_ISL_408514 is not included due to Nil mutation) strain studied in this study in comparison with the reference strain NC_045512.2.

The results illustrated the comparison with reference selection: hCoV-19/Wuhan/WIV04/2019; avoiding unusual mutation and highly diversifying mutation from the reference sequence; Protein: G- glycine, L-Leucine, I- isoleucine, P- Proline, Y- Tyrosine, W- Tryptophan, S- Serine, T-Threonine, C- Cysteine, M- Methionine, N- Asparagine, Q- Glutamine, D- Aspartate, K- Lysine, R- Arginine, H- Histidine, A-Alanine, E- Glutamic acid, F-Phenylalanine (Fig 14).

## Discussion

### SC2r-CoVs in horseshoe bats and pangolin

Animal species which can act as a reservoir, natural host, and intermediate host should be well-known to health authorities. Bats are thought to be the ancestor of SARS-CoV-2. They can also act as reservoirs of several other emerging zoonotic pathogens like Nipah virus, Hendra virus, influenza virus, Ebola virus, rabies virus, and CoVs [38, 39]. More than 200 novel CoVs have been detected in bats over the world [40]. It is believed that MERS-CoV and SARS-CoV may have originated in bats and transmitted through by dromedary camels and civets, respectively [37]. Not only SARS or MERS, other human CoVs like HCoV-NL63 and HCoV-229E have been also originated from bats [41, 42]. We compared six strains of SARS-CoV-2 to 15 closely related members of the SARS-CoV-2 and SARS-CoV family, finding considerable similarity and recombination events among these coronavirus species, particularly among bat-origin members. The most dramatic of them was statistically significant recombination in gene S and ORF1ab from Laos bat BANAL and China bat RaTG13 to SARS-CoV-2. Though SARS-CoV-2 has a 96.10% genetic resemblance with bat CoV RaTG13 (Horseshoe bat/*R*. *affinis*), but no natural infection of SARS-CoV-2 in bat has been reported [17–20]. These findings confirm the theory that the SARS-CoV-2 genome is a chimera formed by the virus recombination with bat (e.g. RatTG13) CoV genomes [32]. The confirmed recombination events in genes S and ORF1ab as well as the SimPlot recombination analysis of genes S, ORF 1ab and N suggest that pangolin is a likely intermediate host of SARS-CoV-2, on its way of transmission from bats to humans which is also supported by some previous study by [32, 43].

Furthermore, *Rhinolophus* bats are the reservoir of SARS-CoV-2 like viruses [44]. Moreover, the SARS-CoV-2 genome has an insertion site between the cleavage site of S1 and S2, found in horseshoe bat (*R*. *malaynus*). Two bat species namely *R*. *affinis* and *R*. *malaynus* can be the ancestral reservoir of SARS-CoV-2. But bats cannot transmit SARS-CoV-2 directly to humans [16]. Two distinct points indicate that bats are not responsible for transmitting the virus to human [45, 46]. Firstly, during the pandemic, local bat species were out and hibernated [45]. Secondly, though other animal species were present in the animal market for sale, bats were not sold at that time [47].

As the *Rhinolophus* bats are distributed in other countries than China, SC2r viruses could be found outside China also. The bat viruses from Cambodia have 92.7% [48] and Japan has 79.06% nucleotide similarity with SARS-CoV-2. The Japanese strain is too distant from SARS-CoV-2 and cannot bind to SARS-CoV-2’s receptor in human cells. So, this strain will not infect humans easily. Most recently four novel SC2r viruses were identified in bat samples from China between May 2019 to November 2020. The virus, RpYN06 detected in *Rhinolophus pusillus* was the closest relative of SARS-CoV-2 with 94.48% sequence identity [49]. Another group of SC2r viruses was detected in Thailand in *R*. *acuminatus* bats having 91.1% genetic resemblance with SARS-CoV-2 [49].

The close phylogenetic relationship between sequences from SC2r viruses from bats from China indicates the virus’ common ancestry. The sequences from the same country have similarities, but there are inter-country variations. So separate clustering of SC2r viruses from bats from different times aligns with the assumption that the viruses are changing over time. A recent study predicted that at least 23 *Rhinolophus* bat species could be found from southern Laos and Vietnam to southern China [49]. Due to the *Rhinolophus* bat’s distribution, the SC2r viruses were detected in the Asian countries rather than in other continents of the world. Thus, Southeast Asia is considered a hotspot of SC2r coronaviruses.

Along with the bat, pangolin was thought to be playing a role in the SARS-CoV-2 transmission [50]. The first SARS-CoV-like CoV was named pangolin-CoV when identified in 2 Malayan pangolin’s carcass on 24 October 2019 [51]. This was supported by pangolin CoV being closely related to SARS-CoV-2 having 85.18 to 89.93% nucleotide resemblances [52]. Along with the bat CoV, pangolin CoV was the second relative of SARS-CoV-2 [52]. However, SARS-CoV-2 cannot be the descendant of pangolin CoV as the genetic variation is too high between them [51]. Furthermore, scientists tested 334 Sunda pangolins seized during illegal trading in Peninsular Malaysia and Sabah between Aug 2009 to Mar 2019. Tests were negative for coronavirus, which reflects that pangolin is no reservoir or intermediate host for SARS-CoV-2, rather they might get infected by humans or any other animal species along with the trade network [53].

Phylogenetic analysis showed that pangolin CoVs have inherent genetic relatedness among them. But there were some variations between samples from Guangdong and Guangxi province of China. Moreover, the Guangdong strain has the closest relation with the human reference strain from Wuhan. So it is of utmost interest to the scientific community to know if pangolin is the natural host [50, 54] or the dead-end host [55]. Future studies are required to prove the link between pangolin and SARS-CoV-2 [54, 56].

### Natural transmission of SARS-CoV-2 from humans to domestic and wild animals

After the emergence of SARS-CoV-2 in December 2019, several animal species were infected naturally. Domestic animals like dog, cat, and wild animals like tiger, lion, gorilla, leopard cat, puma, ferret, cougar, and mink have been infected by SARS-CoV-2 via reverse zoonoses [22, 57, 58]. Animal species that are primarily in contact a humans in settings like a household with pets, zoo, safari park, zoological centers, farms, etc. were infected by humans. We compiled potential animal species which infected naturally and can act as a reservoir or intermediate or susceptible host.

At the beginning of the SARS-CoV-2 outbreak in China, antibodies against SARS-CoV-2 were detected in three different cats owned by three different patients, suggestive of human to cat transmission. Besides, serum samples collected from stray cats or hospital cats showed lower antibody titers. In contrast, SARS-CoV-2 was detected from symptomatic cat’s feces and vomitus in Belgium [59]. Pet cats were also tested positive in New York, the USA which suggesting human-to-cat transmission [60]. In Spain, eight cats were infected by humans [61]. At least one infected human has one pet dog or cat in almost 25% of households in Texas, USA [62]. Cats from the abandoned household of SARS-CoV-2 patients and veterinary clinics showed seropositivity to SARS-CoV-2 in Wuhan, China [16]. So, it is a matter of concern, if the cat becomes infected and remains asymptomatic, then there is a possibility of transmission of the viruses to humans and other animal species including cats, dogs. The first report of a dog infected by SARS-CoV-2 was found on 28 February 2020 in a Pomeranian dog in Hong Kong [63]. The owner of this dog was also tested positive for COVID-19 a few days ago [63]. After that, several dogs were infected without showing any signs in Hong Kong. Those dogs had a history of mutual living places with infected humans [64, 65]. Another dog was infected by its owner in the Netherlands [66].

Several zoo animal species have been infected by SARS-CoV-2 (WAHIS, 2020; WCS, 2020). It has been assumed that the infection might have occurred from pre or asymptomatic animal caretakers in the zoo (WAHIS, 2020). Lion and tigers developed mild respiratory signs like dry cough and wheezing. Nine genomes were identified from these tigers, lions, and keepers [67], but 2 distinct genotypes were found [68]. It has been confirmed that transmission occurred from human to tiger, but the exact route is still unclear [67]. Recently two gorillas at the San Diego Zoo safari park in the USA, have been infected by COVID-19. Gorillas had mild symptoms like coughing and congestion. The zoo authority suspects that an asymptomatic worker who tested positive for SARS-CoV-2 might infect the gorillas [69].

The susceptibility of mink to SARS-CoV-2 has already been established. Minks from two different farms in the Netherlands showed respiratory and gastrointestinal disorders during April 2020 [70]. The mortality rate was estimated as 1.2–2.4% which was higher among pregnant animals. The record of SARS-CoV-2 infected workers is evidence of possible human to animal transmission. However, sequencing the initial data is suggestive in favor of mink to human transmission within mink farms [70]. More than half of human cases (N = 338 cases) in Denmark since June 2020 had a history of working with mink pelting in six factories and two small facilities. It suggests that people involved in farming, culling, and pelting of mink have an increased risk of COVID-19 infection. SARS-CoV-2 infection was detected in 12 feral cats and 2 dogs in the Netherland. The feral cats got the infection from minks but the source of infection for the dogs might be either mink or humans [58, 71]. Infection in wild mink raises the question about SARS-CoV-2 transmission from human to wildlife and its consequences. Whether the infection from human to different animal species was direct or indirect, the main concern should be the wide host range of SARS-CoV-2. The virus gradually mutate and infect new host species gradually [72]. The problem of infecting wildlife is that some wild species like bats, macaque, etc. live-in colonies. If one wild animal gets an infection from humans, it can transmit the virus to an entire colony of animals. Thus, there is the chance of parentally establishing of the virus in the wildlife population, and then there will be reemergence events at the interval. If this happens, it will be very difficult to eradicate the virus. Moreover, vaccination of humans and pets will not be successful also [73].

### Genomic epidemiology of SARS-CoV-2 in animals

SARS-CoV-2 sequences from different animals e.g; dog, cat, tiger, lion, mink, gorilla proved their relation to human sequences [46, 61, 63, 74]. The α-variant/B.1.1.7 and delta variant/B.1.617.2 are common in humans, so it is much predictable to find it in different animal species like dog, lion, tiger, gorilla and cat in some selected countries in Asia, Europe and north America. This VOC and VOI have been transmitted locally through direct or indirect routes [24, 75]. A dog and a cat were infected by the B.1.1.7 after 2 days of their owners being positive for COVID-19 [36]. But this alpha and delta-variant have not affected animal species as they affected the human population severely [76]. Sequences from tigers from the Bronx Zoo, the USA clustered with sequences from tiger keepers. But SARS-CoV-2 which infected lions in that zoo was different from that of tigers. It indicated two different strain introduction in the zoo, one in lion and one in tigers [67]. Moreover, genetic similarities among human and animal strains from European countries like Czech Republic, Germany, Belgium, Switzerland, Netherlands, France, Spain, and Denmark were observed in the phylogeny and further supports the localized human to animal transmission worldwide.

However, there was a profundity of clade O in the dog. Clade O is less prevalent in the humans than in other clades (GISAID). Therefore, we can hypothesize that this clade has a special affinity to the dog. On the other hand, clade GR is very common in the human population throughout the world (GISAID). So, the presence of GR clade in the cat can be related to this species’ availability as pets of humans. Minks were also affected by clade GR and the transmission can be sourced back to humans [70].

The mink variant was detected in humans alongside with mink population of the same countries as the Netherlands, USA, Denmark, Poland. Denmark had a large population of mink where the mink variant did a devastating effect [37]. But interestingly some countries like South Africa, Russia, Switzerland, and England also detected mink variants in humans though they do not have mink farms in the region (https://www.furfreealliance.com/fur-bans/). Human movement can be attributed to the spread of the mink variant in non-farming countries [77]. The record of SARS-CoV-2 infected workers is evidence of possible human to animal transmission. The resemblance between human strains and mink strains indicates transmission of the virus between humans and minks [70]. On the other hand, mink variants in cats from Denmark refer to the spreading of the variant from mink farms’ staff to pet cats [71]. Thus, there are events of SARS-CoV-2 transmission from human to mink, human to cat and dog, and then mink to human.

The common mutations were Y505H and Y453F in different animal species including dog, cat, lion, tiger, and gorilla. The mutation Y453F in spike protein is specific for the mink variant and responsible for displaying a pronounced increase in ACE-2 affinity [25]. The Y453F mutation was found in almost half of the sequenced mink strains. Y453F was detected in Denmark first in the mink population. This variant can transmit to humans from the minks and raised a public health concern [25]. Another commonest mutation was D614G in mink found in almost 95% of minks. D614G mutation is a very common variant in human populations worldwide [78, 79]. Cat and lion strains have some unique mutations which are not present in other animals. So, animal hosts can produce new variants subsequently after mutations. We do not know the specific role of those mutations in animal hosts whether the resultant virus will be more pathogenic or not. Even if the resultant mutant virus is not pathogenic for humans, it could be harmful to respective animal species and threaten the conservation of endangered species. Thus, regular epidemiological and genomic surveillance of animal hosts is recommended to detect and prevent new strains of the virus. We should also consider targeted risk assessment and screening of different wild mustelids, bats, wild canids, and felids in the different zoo and safari parks. Moreover, all animal caretakers should take precautions to prevent viral exposure to wild animal species and vice versa [21, 80, 81].

## Conclusion

A wide range of domestic and wild animal species have been naturally infected by SARS-CoV-2 from the human. Human to animal transmission is limited and sporadic. Animal strains of the virus showed numerous amino acid substitutions though only one variant (mink-variant) originated in animals. Thus, we recommend that infected or suspected human cases not contact domestic/pet animals and wildlife to prevent spillover and spillback events. Furthermore, we recommend vaccinating the pet, zoo, and farmed animals. Moreover, there is a chance of transmission and establishment of the virus in wildlife and the environment. So, continuous targeted serologic and genomic surveillance in animals, especially wildlife, is essential to identify the reservoir hosts and control the regular emergence of the virus in the human population.

## Supporting information

S1 Table SARS-CoV-2 and SARS-CoV-2 related viruses data. (XLSX)

S1 Fig Interspecies clade diversity of SARS-CoV-2. (TIF)
