# Supplementary figures and images for: Spatial epidemiology and genetic diversity of SARS-CoV-2 and related coronaviruses in domestic and wild animals
Source: PLoS One. 2021 Dec 15;16(12):e0260635. doi: 10.1371/journal.pone.0260635 (PMC8673647; doi:10.1371/journal.pone.0260635)

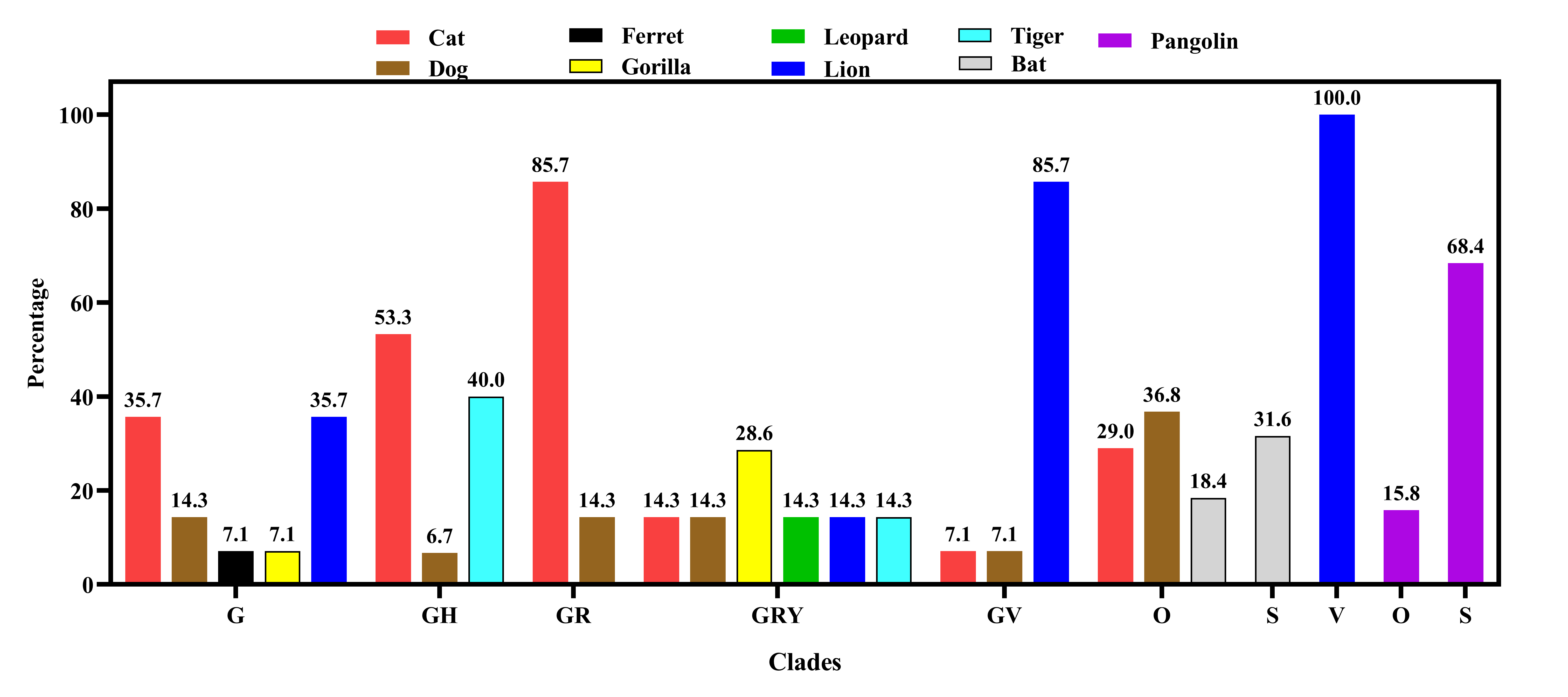

Supplement: S1 Fig — (TIF) [file pone.0260635.s002.tif]
